# Clinical Outcomes of Patients with Combined Idiopathic Pulmonary Fibrosis and Emphysema in the IPF-PRO Registry

**DOI:** 10.1007/s00408-021-00506-x

**Published:** 2022-01-07

**Authors:** Hyun J. Kim, Laurie D. Snyder, Megan L. Neely, Anne S. Hellkamp, David L. Hotchkin, Lake D. Morrison, Shaun Bender, Thomas B. Leonard, Daniel A. Culver, the IPF-PRO™ Registry investigators

**Affiliations:** 1 University of Minnesota, Minneapolis, MN USA; 2 Duke Clinical Research Institute, Durham, NC USA; 3 Duke University Medical Center, Durham, NC USA; 4 Division of Pulmonary, Critical Care & Sleep Medicine, The Oregon Clinic, Portland, OR USA; 5 Boehringer Ingelheim Pharmaceuticals, Inc, Ridgefield, CT USA; 6 Cleveland Clinic, Cleveland, OH USA

**Keywords:** Interstitial lung disease, Hospitalization, Mortality, Pulmonary fibrosis, Respiratory function tests

## Abstract

**Purpose:**

To assess the impact of concomitant emphysema on outcomes in patients with idiopathic pulmonary fibrosis (IPF).

**Methods:**

The IPF-PRO Registry is a US registry of patients with IPF. The presence of combined pulmonary fibrosis and emphysema (CPFE) at enrollment was determined by investigators’ review of an HRCT scan. Associations between emphysema and clinical outcomes were analyzed using Cox proportional hazards models.

**Results:**

Of 934 patients, 119 (12.7%) had CPFE. Compared with patients with IPF alone, patients with CPFE were older (median 72 vs 70 years); higher proportions were current/former smokers (88.2% vs 63.7%), used oxygen with activity (49.6% vs 31.9%) or at rest (30.8% vs 18.4%), had congestive heart failure (13.6% vs 4.8%) and had prior respiratory hospitalization (25.0% vs 16.7%); they had higher FVC (median 71.8 vs 69.4% predicted) and lower DLco (median 35.3 vs 43.6% predicted). In patients with CPFE and IPF alone, respectively, at 1 year, rates of death or lung transplant were 17.5% (95% CI: 11.7, 25.8) and 11.2% (9.2, 13.6) and rates of hospitalization were 21.6% (14.6, 29.6) and 20.6% (17.9, 23.5). There were no significant associations between emphysema and any outcome after adjustment for baseline variables. No baseline variable predicted outcomes better in IPF alone than in CPFE.

**Conclusion:**

Approximately 13% of patients in the IPF-PRO Registry had CPFE. Physiologic characteristics and comorbidities of patients with CPFE differed from those of patients with IPF alone, but the presence of emphysema did not drive outcomes after adjustment for baseline covariates.

**Trial registration:**

ClinicalTrials.gov, NCT01915511; registered August 5, 2013.

**Supplementary Information:**

The online version contains supplementary material available at 10.1007/s00408-021-00506-x.

## Introduction

Idiopathic pulmonary fibrosis (IPF) is a chronic interstitial lung disease associated with progressive decline in lung function, debilitating symptoms, and high mortality [1]. Emphysema is a common comorbidity in patients with IPF [2], but the clinical course of combined pulmonary fibrosis and emphysema (CPFE) continues to be debated. In a case series of 61 patients with CPFE published in 2005, almost half of the patients had pulmonary hypertension (PH) by echocardiogram and median survival was 6.1 years [3]. Subsequent studies have reported mortality in patients with CPFE similar to that in patients with IPF alone [4–6] or worse than in patients with IPF alone [7–10]. Comparing studies is challenging due to the different definitions used for CPFE, variation in the algorithms used to obtain the chest CT scans, and case ascertainment biases.

How emphysema in patients with IPF affects disease progression and survival and how patients with CPFE should be followed remain poorly understood. Compared with patients with IPF alone, patients with CPFE typically have higher forced vital capacity (FVC) but lower diffusing capacity of the lungs for carbon monoxide (DLco) [4]. Different studies have suggested that the measure of lung function that provides the best indicator of prognosis in patients with CPFE may be DLco [11] or forced expiratory volume in 1 s (FEV_1_) [12]. We used data from the IPF-PRO Registry, a multicenter US registry of patients with IPF, to compare the clinical characteristics and outcomes of patients with CPFE and IPF alone and to determine whether baseline measures, such as pulmonary function tests, correlate differently with outcomes in patients with CPFE versus IPF alone.

## Methods

The design of the IPF-PRO Registry has been published [13]. This registry enrolled patients with IPF that was diagnosed or confirmed at the enrolling center in the past 6 months. Patients who were on a lung transplant list or participating in a randomized clinical trial could not be enrolled, but patients could be listed for transplant or enter clinical trials after enrollment. Patients in the registry are followed prospectively while receiving usual care, with follow-up visits scheduled approximately every 6 months. The study was approved by the Duke University Institutional Review Board (Pro00046131). The protocol was approved by the relevant Institutional Review Boards and/or local Independent Ethics Committees prior to patient enrollment at each site listed in the Acknowledgments. All patients provided informed consent.

A total of 1002 patients at 46 sites were enrolled between June 2014 and October 2018. Patients who did not have a high-resolution computed tomography (HRCT) scan, FEV_1_ value and FVC value at enrollment, or had no data recorded after enrollment, were excluded from these analyses. A patient was considered to have CPFE if “clinically significant emphysema” was present on an HRCT scan in the opinion of the investigator, assessed at the time of enrollment into the registry. In the presentation of patient characteristics at enrollment, continuous variables are presented as median (25th percentile, 75th percentile) and categorical variables as number and per cent of patients. Continuous variables were compared between patients with CPFE versus IPF alone using Wilcoxon rank sum tests. Categorical variables were compared using Pearson's chi-squared tests.

Clinical outcomes studied over follow-up were as follows: death; lung transplant; hospitalization (all-cause); death or lung transplant; and death, lung transplant, or hospitalization (all-cause). Cumulative incidence curves (for lung transplant and hospitalization, with death as a competing risk) and Kaplan–Meier event curves (for other outcomes) were estimated using all available follow-up data. Event rates at 1 year were estimated. Associations between the presence of emphysema and each outcome were assessed using univariable and multivariable Cox proportional hazards models. For lung transplant and hospitalization, Fine and Gray models were used, with death considered as a competing risk. In addition, associations between the presence of emphysema based on a broader definition (clinically significant emphysema on HRCT and/or FEV_1_/FVC < 0.7 at enrollment) and each outcome were assessed using the same modeling approach. The univariable model included the presence of emphysema as the only covariate. Multivariable models included emphysema and variables identified as being associated with the outcome in the entire registry cohort (Supplementary Table 1).

Associations between the GAP (gender-age-lung physiology) score [14] and the composite physiologic index (CPI) [15] and outcomes were assessed using multivariable models. The association between baseline variables and disease severity metrics and outcomes in patients with CPFE versus IPF alone were assessed using interaction tests. A significant interaction indicated that the relationship between the baseline variable and the outcome was different depending on whether the patient had emphysema.

When performing the modeling, the linearity assumption was assessed for all continuous covariates using restricted cubic splines, and transformations such as piecewise linear splines were used to accommodate for violations where necessary. The proportional hazards assumption was checked for CPFE in all models using the Kolmogorov-type supremum test, and no violations were found. Missing data were handled using multiple imputation: the missing data were filled in five times to generate five complete data sets using the Full Conditional Specification method; each complete data set was analyzed using standard statistical analyses; the results from the five complete datasets were averaged to generate the final inferential results.

The proportion of patients with new-onset PH (as reported by the investigator) during follow-up was assessed descriptively.

## Results

### Patients

Of 934 patients in the analysis cohort, 119 (12.7%) had CPFE at enrollment based on the presence of clinically significant emphysema on an HRCT scan, in the opinion of the investigator. Compared with patients with IPF alone, patients with CPFE were older (median age 72 vs 70 years) and higher proportions were current/former smokers (88.2% vs 63.7%), used oxygen with activity (49.6% vs 31.9%), used oxygen at rest (30.8% vs 18.4%), had congestive heart failure (13.6% vs 4.8%), and had a prior respiratory hospitalization (25.0% vs 16.7%) (Table 1). Patients with CPFE had a higher median FVC (71.8 vs 69.4% predicted) and lower median DLco (35.3 vs 43.6% predicted) than patients with IPF alone. The distribution of pulmonary function tests, GAP scores, and CPI at enrollment are presented in Supplementary Figs. 1 and 2. Patients with CPFE had worse St. George’s Respiratory Questionnaire (SGRQ) activity scores (median 66.2 vs 54.5) and better Cough and Sputum Assessment Questionnaire (CASA-Q) cough impact (median 87.5 vs 75.0) and symptoms (median 66.7 vs 58.3) domain scores than patients with IPF alone (Table 2). When CPFE was defined using a broader definition of clinically significant emphysema on HRCT and/or FEV_1_/FVC < 0.7, 149 patients (16.0%) had CPFE at enrollment.

**Table 1:** Patient characteristics at enrollment into the IPF-PRO Registry by presence of emphysema

	CPFE (*n* = 119)	IPF alone (*n* = 815)	*P*-value
Age, years	72 (67, 76)	70 (65, 75)	0.011
Male	92 (77.3)	599 (73.5)	0.38
White	108 (93.9)	764 (95.7)	0.38
Smoking history			<0.001
Current	3 (2.5)	13 (1.6)	
Former	102 (86.4)	506 (62.1)	
Never	13 (11.0)	296 (36.3)	
Hospitalization in prior 12 months	37 (33.0)	220 (28.0)	0.27
Respiratory hospitalization in prior 12 months	28 (25.0)	131 (16.7)	0.031
Prior diagnosis of IPF (before referral to enrolling center)	50 (42.0)	354 (43.5)	0.75
Diagnostic criteria for IPF^a^			0.91
Definite IPF	79 (66.4)	536 (65.8)	
Probable IPF	28 (23.5)	204 (25.0)	
Possible IPF	12 (10.1)	75 (9.2)	
GAP score [14]	4.5 (4.0, 5.0)	4.0 (3.0, 5.0)	0.039
GAP stage [14]			0.12
I	22 (22.0)	222 (31.0)	
II	56 (56.0)	376 (52.6)	
III	22 (22.0)	117 (16.4)	
CPI^15^	55.7 (48.3, 62.6)	52.1 (44.6, 59.4)	0.005
FEV_1_% predicted	77.9 (68.1, 94.5)	77.2 (65.0, 88.4)	0.089
FVC, % predicted	71.8 (63.4, 90.8)	69.4 (58.8, 79.0)	0.001
FEV_1_/FVC, %	79.5 (75.0, 85.5)	82.9 (78.4, 86.9)	<0.001
FEV_1_/FVC < 70%	16 (13.4)	30 (3.7)	<0.001
DLco, % predicted	35.3 (26.6, 44.1)	43.6 (34.0, 52.2)	<0.001
History of gastro-esophageal reflux disease	63 (53.4)	460 (56.7)	0.50
History of coronary artery disease	40 (34.2)	237 (29.2)	0.27
History of obstructive sleep apnea	39 (33.1)	221 (27.3)	0.19
History of atrial fibrillation or flutter	15 (12.7)	82 (10.1)	0.39
History of PH	12 (10.3)	52 (6.4)	0.13
History of congestive heart failure	16 (13.6)	39 (4.8)	<0.001
Oral steroid	19 (18.6)	85 (11.5)	0.040
Bronchodilator	56 (54.4)	204 (27.6)	<0.001
Pulmonary vasodilator	5 (4.9)	18 (2.4)	0.16
Pirfenidone	35 (29.4)	255 (31.3)	0.68
Nintedanib	37 (31.4)	184 (22.6)	0.036
Oxygen with activity	57 (49.6)	254 (31.9)	<0.001
Oxygen at rest	36 (30.8)	146 (18.4)	0.002

Data are median (25th, 75th percentile) or n (%). ^a^According to 2011 ATS/ERS/JRS/ALAT diagnostic guidelines [20]

**Table 2:** Patient-reported outcomes at enrollment into the IPF-PRO Registry by presence of emphysema

	CPFE (*n* = 119)	IPF alone (*n* = 815)	*P*-value
SGRQ total score	41.8 (27.3, 53.7)	39.5 (25.1, 53.7)	0.34
SGRQ activity score	66.2 (47.6, 79.1)	54.5 (37.4, 72.8)	0.004
SGRQ impact score	26.4 (14.3, 40.0)	26.1 (14.2, 42.1)	0.96
SGRQ symptoms score	39.8 (26.2, 59.3)	44.5 (30.8, 61.7)	0.10
CASA-Q cough impact domain	87.5 (68.8, 96.9)	75.0 (56.3, 93.8)	<0.001
CASA-Q cough symptoms domain	66.7 (50.0, 83.3)	58.3 (41.7, 75.0)	<0.001
EQ-5D index score	0.8 (0.7, 0.9)	0.8 (0.7, 1.0)	0.083
EQ-5D VAS score	70.0 (60.0, 85.0)	75.0 (61.0, 85.0)	0.052
SF-12 mental component summary	53.3 (45.1, 60.2)	54.1 (45.8, 59.2)	0.92
SF-12 physical component summary	37.5 (32.2, 43.6)	39.4 (31.1, 46.6)	0.29

Data are median (25th, 75th percentile). SGRQ. St. George’s Respiratory Questionnaire. CASA-Q, Cough and Sputum Assessment Questionnaire. EQ-5D, EuroQoL 5-D index score. VAS, Visual analog scale. SF-12, Short Form-12

### Pulmonary Hypertension

Among patients with available data, 12 of 117 patients (10.3%) with CPFE and 52 of 810 patients (6.4%) with IPF alone had a history of PH at enrollment (*p* = 0.13). New-onset PH was reported during follow-up in 4 of 105 patients (3.8%) with CPFE and 43 of 758 (5.7%) patients with IPF alone at enrollment.

### Associations Between CPFE and Outcomes

**Fig. 1:**
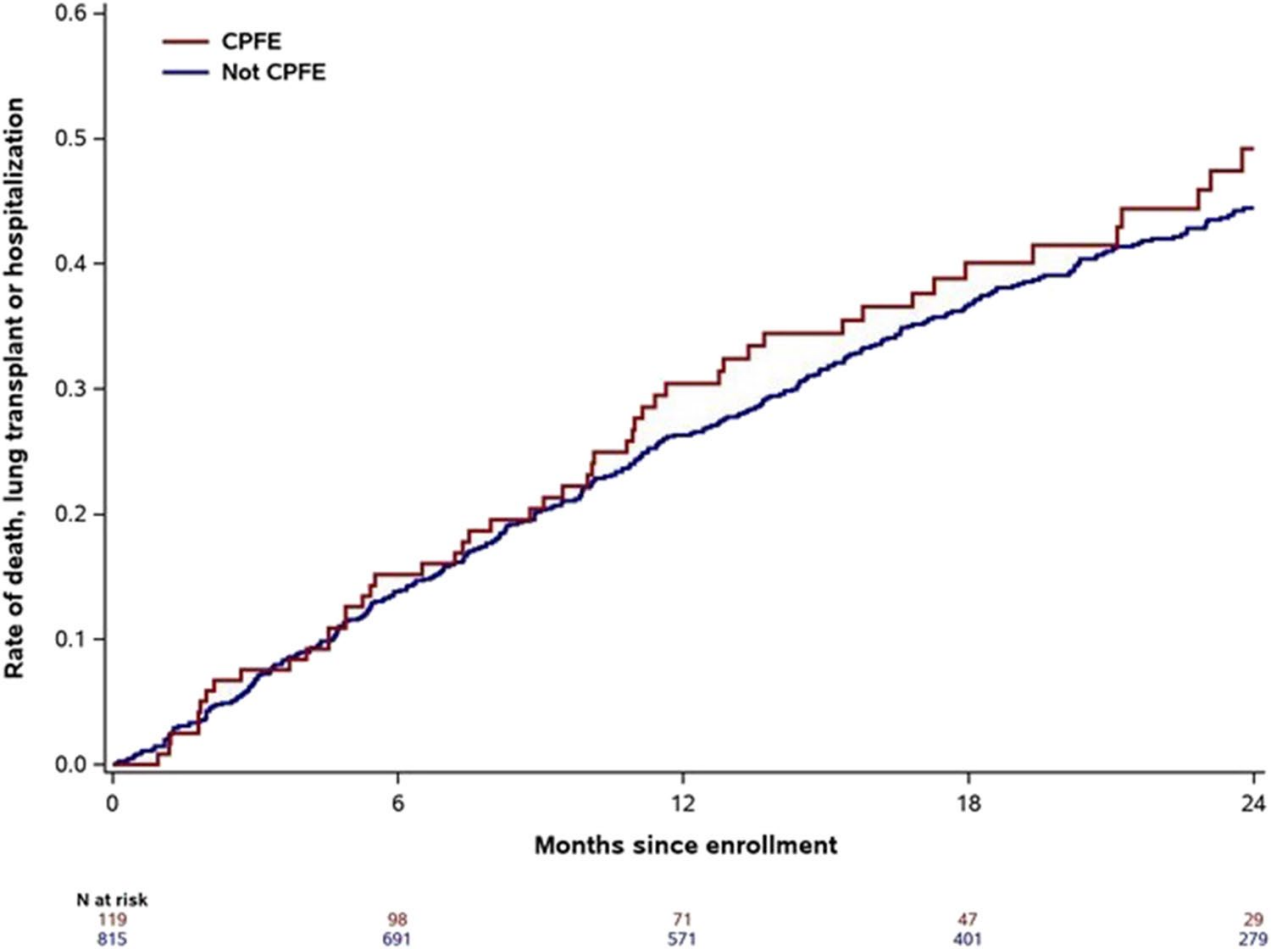
Kaplan–Meier plot of time to death, lung transplant, or hospitalization by presence of emphysema at enrollment

**Fig. 2:**
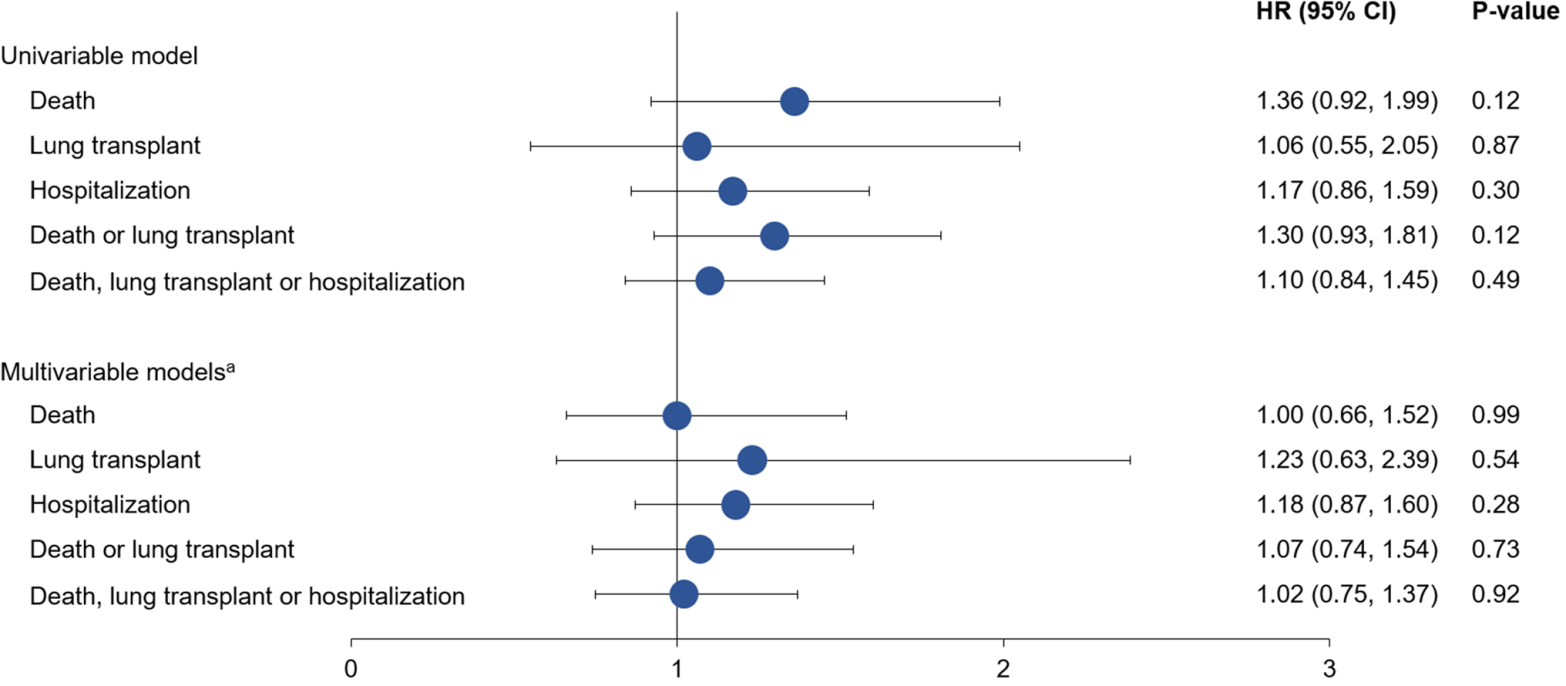
Associations between emphysema at enrollment and clinical outcomes.^a^Adjustment variables included in the models were as follows (all at enrollment): age, BMI, FVC % predicted, DLco % predicted, oxygen use at rest, and history of coronary artery disease or heart failure for time to death. Age, BMI, FEV_1_ % predicted, FVC % predicted, DLco % predicted, oxygen use at rest, oxygen use with activity, and prior diagnosis of IPF (before referral to enrolling center) for time to lung transplant. BMI, FEV_1_ % predicted, and oxygen at rest for time to hospitalization. Age, BMI, FEV_1_ % predicted, FVC % predicted, DLco % predicted, oxygen use at rest, oxygen use with activity, history of coronary artery disease or heart failure, and prior diagnosis of IPF (before referral to enrolling center) for time to death or lung transplant and for time to death, lung transplant or hospitalization

For all the outcomes studied, estimates for event rates at 1 year were numerically higher in patients with CPFE versus IPF alone at enrollment (Table 3; Fig. 1; Supplementary Figs. 3–6). However, there were no statistically significant associations between the presence of emphysema at enrollment and any outcome evaluated in univariable or multivariable models (Fig. 2). Similarly, there were no significant associations between the presence of emphysema at enrollment and any outcome evaluated when the broader definition of emphysema was used (Supplementary Table 2 and Supplementary Fig. 7).

**Table 3:** Kaplan–Meier estimated event rates at 1 year by presence of emphysema at enrollment

	CPFE	IPF alone
Death		
Cumulative event count at 1 year	30	194
Event rate at 1 year, % (95% CI)	13.1 (8.1, 20.9)	7.8 (6.1, 9.9)
Lung transplant		
Cumulative event count at 1 year	10	74
Event rate at 1 year, % (95% CI)	5.1 (2.1, 11.7)	3.8 (2.6, 5.4)
Hospitalization		
Cumulative event count at 1 year	46	301
Event rate at 1 year, % (95% CI)	21.6 (14.6, 29.6)	20.6 (17.9, 23.5)
Death or lung transplant		
Cumulative event count at 1 year	40	268
Event rate at 1 year, % (95% CI)	17.5 (11.7, 25.8)	11.2 (9.2, 13.6)
Death, lung transplant, or hospitalization		
Cumulative event count at 1 year	58	417
Event rate at 1 year, % (95% CI)	30.4 (22.9, 39.7)	26.3 (23.4, 29.5)

For each outcome, the time to the first event was analyzed

### Associations Between Baseline Variables and Outcomes in Patients with CPFE Versus IPF Alone

There were no significant interactions between baseline variables and CPFE status for the outcomes of death, or death or lung transplant (data not shown). There were no significant interactions between baseline FVC, FEV_1_, or DLco and CPFE status for any of the outcomes. For the outcome of hospitalization, evidence of an interaction was found between CPFE status and oxygen use at rest (interaction *p*-value = 0.026) and age (interaction *p*-value = 0.032). In patients with CPFE, the risk of hospitalization was higher among those who were using oxygen at rest than in those who were not (HR 2.43 [95% CI: 1.39, 4.26]). Oxygen use at rest was not associated with a higher risk of hospitalization among patients with IPF alone (HR 1.16 [95% CI: 0.84, 1.60]). In patients with CPFE, among patients aged > 75 years, the risk of hospitalization was lower for older patients (HR 0.81 [95% CI: 0.67, 0.99] per 1-year increase above 75 years). Among patients with IPF alone, in the same age group, age was not associated with risk of hospitalization (HR 1.03 [95% CI: 0.98, 1.09] per 1-year increase above 75 years). Among patients aged ≤ 75 years, age was not associated with risk of hospitalization in either group (CPFE: HR 1.03 [95% CI: 0.97, 1.10] per 1-year increase up to 75 years; IPF alone: HR 1.00 [95% CI: 0.98, 1.02] per 1-year increase up to 75 years).

### Associations Between GAP Score and CPI and Outcomes in Patients with CPFE Versus IPF Alone

Overall, the risk of death, or death or lung transplant, was higher among patients with a higher GAP score. Although there was no significant interaction between GAP score and CPFE for any outcome, the risk of hospitalization was numerically higher in patients who had a higher GAP score in patients with CPFE but not in patients with IPF alone (Table 4).

**Table 4:** Association between GAP score and CPI at enrollment and clinical outcomes

	Death	Lung transplant	Hospitalization	Death or lung transplant	Death, lung transplant, or hospitalization
*GAP score*					
Overall association					
HR (95% CI) per 1-point increase	1.44 (1.28, 1.62)	0.99 (0.79, 1.22)	1.00 (0.91, 1.10)	1.23 (1.10, 1.38)	1.08 (0.99, 1.18)
*p*-value	<0.001	0.89	0.99	<0.001	0.073
*P*-value for interaction between GAP score and presence of emphysema	1.00	–	0.070	0.78	0.16
HR (95% CI) per 1-point increase in patients with CPFE	–	–	1.24 (0.97, 1.59)	–	–
HR (95% CI) per 1-point increase in patients with IPF alone	–	–	0.98 (0.88, 1.08)	–	–
*CPI*					
Overall association					
HR (95% CI) per 5-point increase	1.38 (1.26, 1.51) ^a^	1.39 (1.23, 1.57) ^a^	1.01 (0.95, 1.07)	1.40 (1.29, 1.51) ^a^	1.18 (1.11, 1.26) ^a^
* p*-value	< 0.001	< 0.001	0.73	< 0.001	< 0.001
*P*-value for interaction between CPI and presence of emphysema	0.75	–	0.060	0.33	0.071
HR (95% CI) per 5-point increase in patients with CPFE	–	–	1.18 (0.99, 1.40)	–	1.37 (1.15, 1.62)
HR (95% CI) per 5-point increase in patients with IPF alone	–	–	0.99 (0.94, 1.05)	–	1.16 (1.09, 1.24)

^a^HR for CPI > 45. Interaction tests were not carried out for lung transplant due to the small number of events among patients with CPFE

Overall, the risk of death, lung transplant, death or lung transplant, or death, lung transplant or hospitalization was higher with increasing CPI when CPI was > 45. Although there was no significant interaction between CPI and CPFE for any outcome, the risk of hospitalization, or death, lung transplant, or hospitalization, was numerically higher in patients with a higher CPI in patients with CPFE but not in patients with IPF alone (Table 4).

## Discussion

The presence of emphysema in patients with IPF is often viewed as a unique phenotype, with worse prognosis and a high likelihood of concomitant PH. We evaluated this impression by analyzing a large real-world prospective multicenter dataset of patients with IPF. Of 934 subjects, 119 were deemed to have significant emphysema, yielding a prevalence of CPFE of 13%. Using a broader definition that included the FEV_1_/FVC ratio, 16% of the registry population had CPFE. We found that the presence of emphysema did not independently confer risk for poor outcomes, after adjustment for a comprehensive set of baseline variables.

The prevalence of CPFE in our population was lower than in several prior studies [2, 5, 7–9, 16], but not all prior studies [4]. A lower prevalence of smoking, the requirement for emphysema to be regarded as “clinically significant”, and recruitment of a broader population including patients with less severe disease are possible explanations for the relatively low prevalence of CPFE in our study. Similar to prior reports, in our study patients with CPFE tended to have more severe disease at baseline, as evidenced by a greater proportion using supplemental oxygen and worse pulmonary function test parameters, compared with patients without emphysema. Additionally, patients with emphysema were older, had more comorbidities, and had a higher likelihood of prior respiratory hospitalization compared with those without emphysema.

Several single-center reports have suggested worse outcomes in patients with emphysema [7–10] but in most of these studies, the impact of CPFE on mortality did not persist after multivariable adjustment. In other studies, the presence of emphysema was not associated with mortality [2, 4, 5]. A meta-analysis including 13 studies found no significant difference in mortality between patients with IPF alone and CPFE at 1, 3, or 5 years [6]. It is possible that findings of increased mortality in patients with CPFE compared with those with IPF alone have been driven largely by confounding variables, including age, tobacco exposure, smoking-related comorbidities, and degree of fibrosis [7, 10]. Our study provides further evidence that CPFE is not an independent risk factor for poor outcomes in patients with IPF. There were no significant interactions between baseline variables and CPFE status for any outcome studied: these variables had the same predictive utility in patients with CPFE as IPF alone.

Patients with CPFE had worse quality of life, as measured by the SGRQ activity score, than those with IPF alone. The impact on physical activity associated with emphysema may in part be indicative of greater physiologic impairment. However, the effect of cough on health-related quality of life, as assessed using the CASA-Q cough domains, was less in patients with CPFE, with between-group medians approximating the minimal important difference for patients with chronic obstructive pulmonary disease [17]. The reasons for the lower impact of cough in the patients with CPFE are unclear, but the more frequent use of inhaler therapy and systemic corticosteroids may be partly responsible.

Although FVC is often relatively preserved in patients with CPFE compared with IPF alone, we found no evidence that baseline FVC or DLco were less useful prognostic markers in the CPFE group. It is possible that changes in FVC or DLco may be differentially prognostic in the CPFE population, but we did not evaluate this. Similarly, disease severity metrics, including GAP score and CPI, did not predict the likelihood of death or lung transplant differently in patients with CPFE than IPF alone. There were non-significant trends for a greater likelihood of hospitalization with worse GAP or CPI values in patients with CPFE than IPF alone (interaction *p*-values 0.07 and 0.06, respectively). Although not conclusive, these data suggest the possibility that CPFE should be assessed as a covariate when using hospitalization as an outcome predicted by GAP or CPI score. Given the similar point estimates for risk, it is possible that worsening physiology, rather than age or sex, account for the increased risk of hospitalization in those with worse scores.

An association between emphysema and PH has been observed in patients with IPF [3, 7, 18]. While we found that PH was numerically more frequent in patients with CPFE than in patients with IPF alone, the prevalence of recognized PH in our CPFE population was only 12%. This could be due to under-diagnosis, but other factors may account for the discrepant findings. For example, because of the prevailing belief that CPFE and PH are strongly associated, practicing clinicians may test more aggressively for PH in patients with CPFE, which may have led to higher estimates of its prevalence in prior reports. In combination with pulmonary fibrosis, emphysema markedly reduces DLco, which may also lead to earlier evaluation for PH [5]. Additionally, the combined effects of parenchymal destruction from pulmonary fibrosis and emphysema result in defects in a higher percentage of the lung. When the percentage of total lung volume is taken into account, the presence of emphysema per se is not associated with PH in patients with IPF [19].

Our study has limitations. Case definition was based solely on the investigator’s appraisal of “clinically significant” emphysema on the baseline HRCT scan. Several prior studies [4, 5, 7, 8, 16] have used quantitative assessments of emphysema. The lower prevalence of emphysema in our study compared with most prior studies suggests that we included only more severe/apparent emphysema, which may have resulted in a higher chance of finding a statistical association with worse outcomes. Instead, we found no independent association between emphysema and important outcomes. We acknowledge that the broader definition that we used for CPFE (*i.e.,* clinically significant emphysema on HRCT and/or FEV_1_/FVC < 0.7) may have been confounded by conditions other than emphysema that cause airway obstruction (*e.g*., asthma). It is possible that we underestimated the prevalence of PH since there was no pre-specified assessment for PH. Even if a significant proportion of PH cases were missed, the prevalence of PH in our study is substantially lower than prior reports and it is likely that the majority of unrecognized PH would be classified as mild.

## Conclusion

Our data challenge the prevailing conception of CPFE; they suggest that confounding variables such as age or complications of smoking may account for the high mortality associated with CPFE in prior reports. Whether CPFE is a unique biologic entity or simply represents the additive effect of two superimposed diseases requires further study.

A plain language summary of this article is available at www.usscicomms.com/respiratory/kim/IPF-PROCPFE.

## Supplementary Information

Below is the link to the electronic supplementary material

Supplementary file1 (DOCX 333 kb)

## Data Availability

The datasets analyzed during the current study are not publicly available, but are available from the corresponding author on reasonable request.
