# Supplementary material for: Clinical Outcomes of Patients with Combined Idiopathic Pulmonary Fibrosis and Emphysema in the IPF-PRO Registry
Source: Lung. 2022 Jan 7;200(1):21–9. doi: 10.1007/s00408-021-00506-x (PMC8881259; doi:10.1007/s00408-021-00506-x)
Supplement: Supplementary file 1 — Supplementary file1 (DOCX 333 kb) [file 408_2021_506_MOESM1_ESM.docx]

**Supplementary material**

Supplementary Table 1. Adjustment models for each outcome

| **Variable** | **Death** | **Lung transplant** | **Hospitalization** | **Death or**  **lung transplant** | **Death, lung transplant,**  **or hospitalization** |
| --- | --- | --- | --- | --- | --- |
| Age | NL | NL |  | NL | NL |
| Body mass index | NL | L | L | NL | NL |
| FEV_1_ % predicted |  | L | L | L | L |
| FVC % predicted | L | L |  | L | L |
| DLCO % predicted | L | L |  | L | L |
| Oxygen with activity |  | X |  | X | X |
| Oxygen at rest | X | X | X | X | X |
| Coronary artery disease or heart failure | X |  |  | X | X |
| Prior diagnosis of IPF (before referral to enrolling center) |  | X |  | X | X |

NL, continuous adjustment covariate with a non-linear relationship with the outcome. L, continuous adjustment covariate with a linear relationship with the outcome. X, binary categorical adjustment covariate.

Supplementary Table 2. Kaplan–Meier estimated event rates at 1 year by presence of emphysema at enrollment (based on clinically significant emphysema on HRCT and/or FEV_1_/FVC <0.7)

|  | **CPFE** | **IPF alone** |
| --- | --- | --- |
| Death, n |  |  |
| Cumulative event count at 1 year | 37 | 187 |
| Event rate at 1 year, % (95% CI) | 12.4 (8.0, 19.0) | 7.7 (6.0, 9.8) |
| Lung transplant, n |  |  |
| Cumulative event count at 1 year | 10 | 74 |
| Event rate at 1 year, % (95% CI) | 4.0 (1.7, 9.3) | 3.9 (2.7, 5.6) |
| Hospitalization, n |  |  |
| Cumulative event count at 1 year | 60 | 287 |
| Event rate at 1 year, % (95% CI) | 24.7 (18.0, 31.9) | 20.0 (17.3, 22.9) |
| Death or lung transplant, n |  |  |
| Cumulative event count at 1 year | 47 | 261 |
| Event rate at 1 year, % (95% CI) | 15.9 (10.9, 22.9) | 11.3 (9.3, 13.8) |
| Death, lung transplant, or hospitalization, n |  |  |
| Cumulative event count at 1 year | 75 | 400 |
| Event rate at 1 year, % (95% CI) | 32.3 (25.3, 40.6) | 25.8 (22.8, 29.0) |

For each outcome, the time to the first event was analyzed.

Supplementary Figure 1. Distribution of pulmonary function tests by presence of emphysema at enrollment


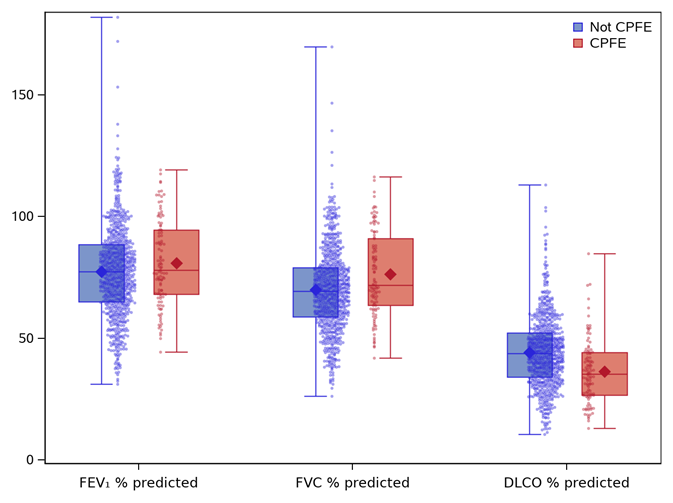


The top and bottom edges of the box denote the interquartile range, the line inside the box the median, the diamond the mean and the whiskers the minimum and maximum values. The dots denote the observed values.

Supplementary Figure 2. Distribution of GAP score and CPI by presence of emphysema at enrollment


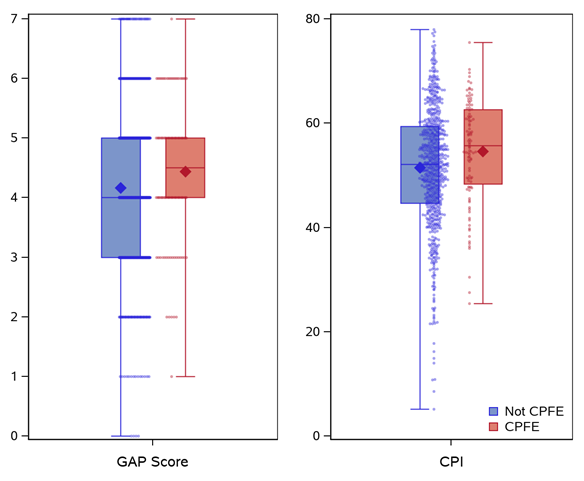


The top and bottom edges of the box denote the interquartile range, the line inside the box the median, the diamond the mean and the whiskers the minimum and maximum values. The dots denote the observed values.

Supplementary Figure 3. Time to death or lung transplant by presence of emphysema at enrollment


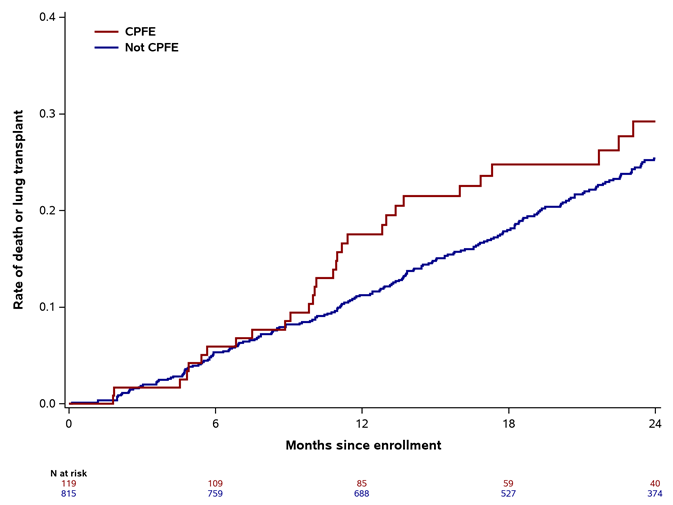


Supplementary Figure 4. Time to death by presence of emphysema at enrollment


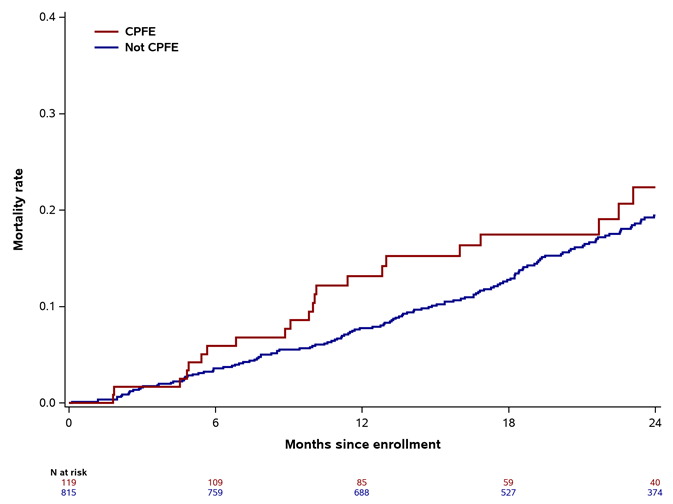


Supplementary Figure 5. Time to lung transplant by presence of emphysema at enrollment


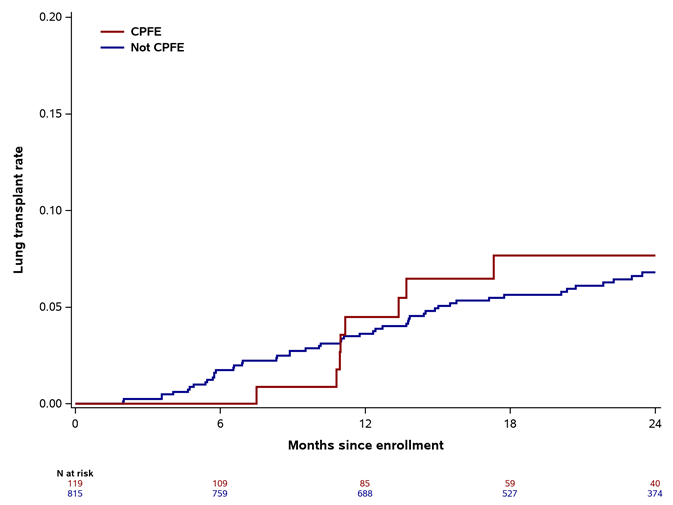


Supplementary Figure 6. Time to hospitalization by presence of emphysema at enrollment


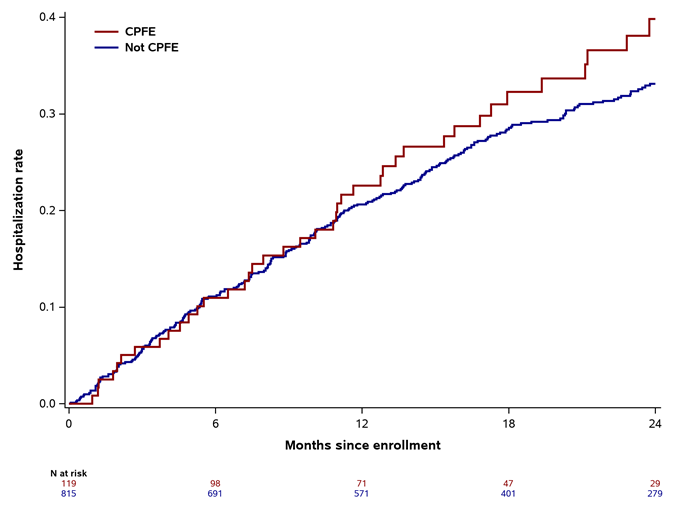


Supplementary Figure 7. Association between emphysema at enrollment (based on clinically significant emphysema on HRCT and/or FEV_1_/FVC <0.7) and clinical outcomes


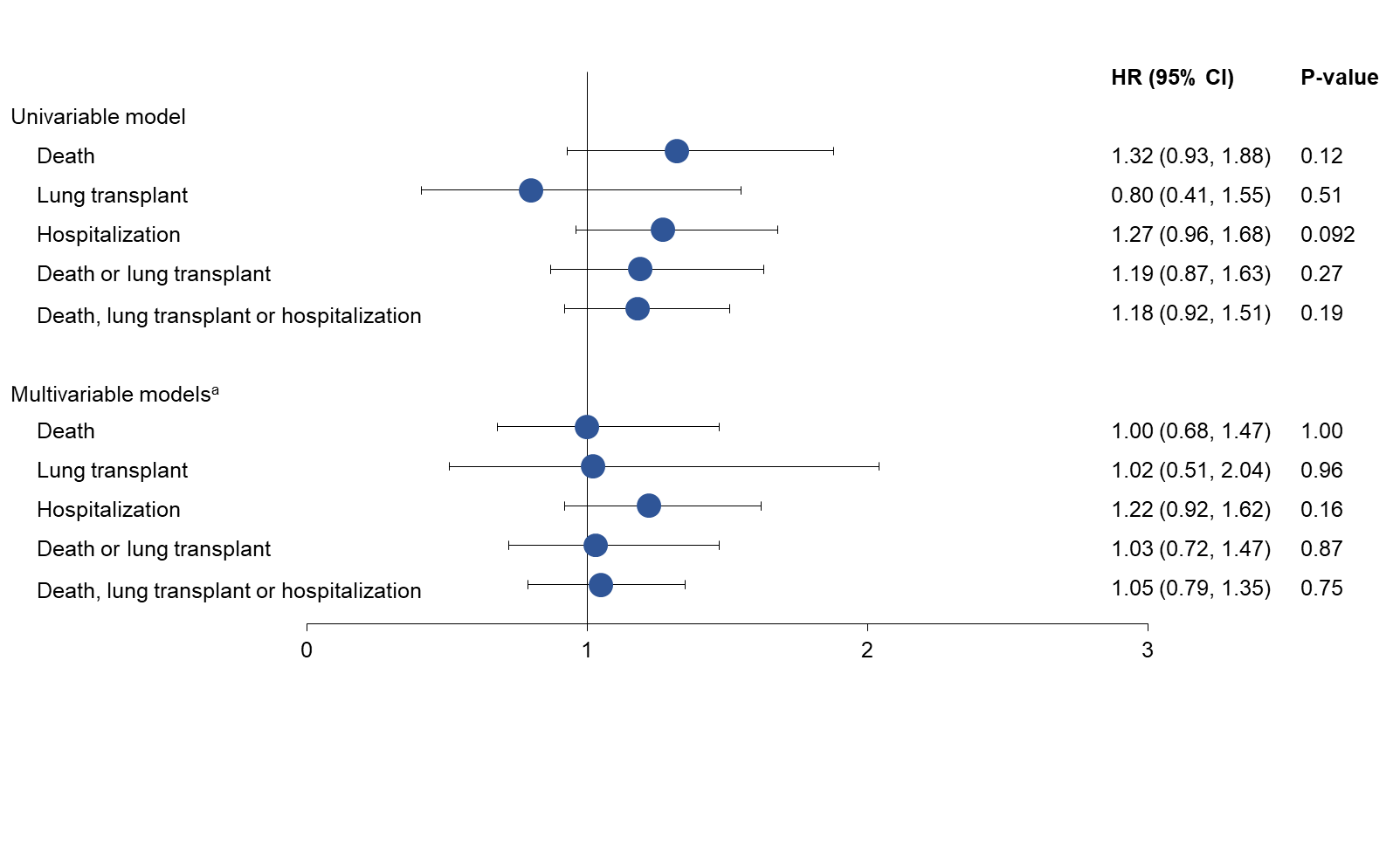


^a^Adjustment variables included in the models were as follows (all at enrollment): age, BMI, FVC % predicted, DLco % predicted, oxygen use at rest, and history of coronary artery disease or heart failure for time to death. Age, BMI, FEV_1_ % predicted, FVC % predicted, DLco % predicted, oxygen use at rest, oxygen use with activity, and prior diagnosis of IPF (before referral to enrolling center) for time to lung transplant. BMI, FEV_1_ % predicted, and oxygen at rest for time to hospitalization. Age, BMI, FEV_1_ % predicted, FVC % predicted, DLco % predicted, oxygen use at rest, oxygen use with activity, history of coronary artery disease or heart failure, and prior diagnosis of IPF (before referral to enrolling center) for time to death or lung transplant and for time to death, lung transplant or hospitalization.
